# Aseptic meningitis in Kikuchi-Fujimoto Disease - Rare manifestation of a rare disease

**DOI:** 10.1016/j.ensci.2022.100429

**Published:** 2022-10-10

**Authors:** Masooma Hashmat, Sadaf Iftikhar, Muhammad Aemaz Ur Rehman, Aqeeb Ur Rehman, Hareem Farooq, Abyaz Asmar, Muhammad Ebaad Ur Rehman

**Affiliations:** a Department of Medicine, Mayo Hospital, King Edward Medical University, Lahore 54000, Pakistan; b Assistant Professor of Neurology, Mayo Hospital, King Edward Medical University, Lahore 54000, Pakistan; c Department of Neurology, Mayo Hospital, King Edward Medical University, Lahore 54000, Pakistan; d Allama Iqbal Medical College, Pakistan

**Keywords:** Aseptic meningitis, Histiocytic lymphadenitis, Kikuchi-Fujimoto Disease, Necrotizing lymphadenitis

## Abstract

Kikuchi-Fujimoto Disease (KFD) is a rare disease marked by necrotizing lymphadenitis, often presenting as unilateral cervical lymphadenopathy, along with various extranodal manifestations such as fever, skin rash, hepatosplenomegaly, and arthritis, etc. KFD is thought to be secondary to either a viral infection or an autoimmune process, however, evidence in favor of both models is scarce and non-definitive. We report a case of a young female who presented with persistent high-grade fever, bilateral cervical and axillary lymphadenopathy, and leukopenia. Excisional biopsy of affected lymph nodes revealed well-circumscribed foci of necrosis with karyorrhectic debris and scattered fibrin deposits characteristic of KFD. The patient was promptly initiated on non-steroidal anti-inflammatory drugs (NSAIDs), however, despite an early improvement in symptoms, the patient soon developed aseptic meningitis, a rare neurological complication of KFD. Intravenous followed by oral corticosteroid therapy reported a good prognosis, with no observable residual neurological deficits. Knowledge of the disease and its complications significantly helped in the avoidance of unnecessary investigations and a delay in treatment.

## Highlights

• Central nervous system involvement may be seen in Kikuchi Fujimoto Disease (KFD).
• Aseptic meningitis is a rare neurological complication of KFD.
• It may present after the resolution of KFD symptoms.
• Prompt treatment and recognition holds favorable prognosis.

## 1 Introduction

Kikuchi Fujimoto Disease (KFD), also known as histiocytic necrotizing lymphadenitis, is a rare self-limiting disorder characterized primarily by posterior cervical lymphadenopathy (usually unilateral) and fever [1]. Although the exact etiology is unclear, several pathogenic models have either described it as a direct sequela of a viral infection [2] or as an autoimmune condition that develops secondary to a viral infection [2]. Moreover, KFD has also been documented as a complication of several systemic autoimmune conditions, such as systemic lupus erythematosus (SLE), Graves' disease, polymyositis, rheumatoid arthritis, etc. [3]. Although KFD has historically been known to predominantly affect females, recent studies have also reported equal or lesser incidence in females as compared to males [4]. Presentation of this disease is highly variable and includes, but is not limited to, fever, leukopenia, rash, and lymphadenopathy [6]. Histopathological features are by far the most important diagnostic criteria, showing lymph node necrosis with karyorrhexis, surrounded by histiocytes, characteristically without neutrophil or plasma cell infiltrate [5]. Treatment is mostly supportive (NSAIDs, antipyretics) in mild cases while corticosteroids are effective in severe cases [6]. This case report describes a 28-year-old female who was histologically diagnosed with KFD and initially treated with NSAIDs, but later presented with aseptic meningitis, an extremely rare neurological complication of this disease. Through this case report, we intend to review the nervous system involvement in KFD and reiterate that the knowledge of these complications can prevent significant patient morbidity and mortality.

## 2 Case report

### 2.1 Early presentation

A 28-year-old married female presented with persistent high-grade fever for two weeks, associated with rigors, chills, and severe body ache followed by painful bilateral swellings in the neck and axilla. The patient reported no headache at this stage of the presentation. The patient was normotensive, nondiabetic, and had no personal history of any other comorbid condition. Her body temperature was 103 °F, respiratory rate 20/min, blood pressure 100/70 mmHg, and pulse rate 120/min. Bilateral tender cervical and axillary lymphadenopathy were the only positive findings on physical examination. There was no nuchal rigidity, the Kernig sign was negative, and no other finding was clinically suggestive of meningitis. A complete blood count (CBC) showed leukopenia with a white cell count (WBC) of 2.6 × 10^9^/L (4–11 × 10^9^/L), microcytic hypochromic anemia with hemoglobin of 10.4 g/dl, and a normal platelet count 321 × 10^9^/L. Kidney and liver functions were unremarkable and anti-nuclear antibodies (ANA) were negative. She was treated with multiple courses of intravenous broad-spectrum antibiotics. Computerized Tomography (CT) scans of the neck, chest, abdomen, and pelvis were performed to aid in a diagnosis of exclusion and were all unremarkable.

**Fig. 1:**
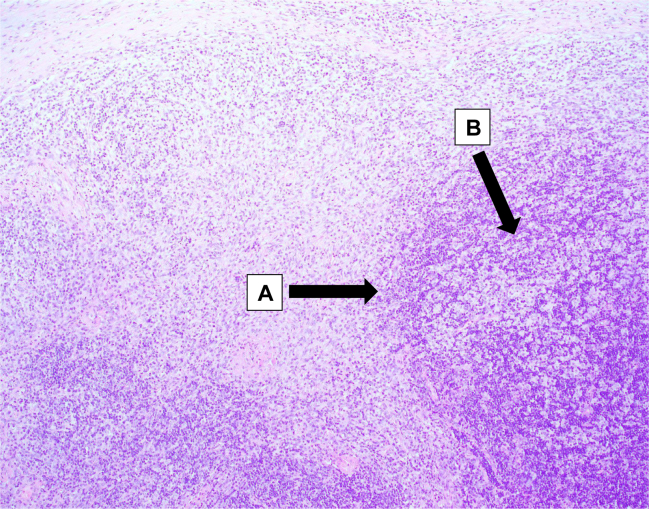

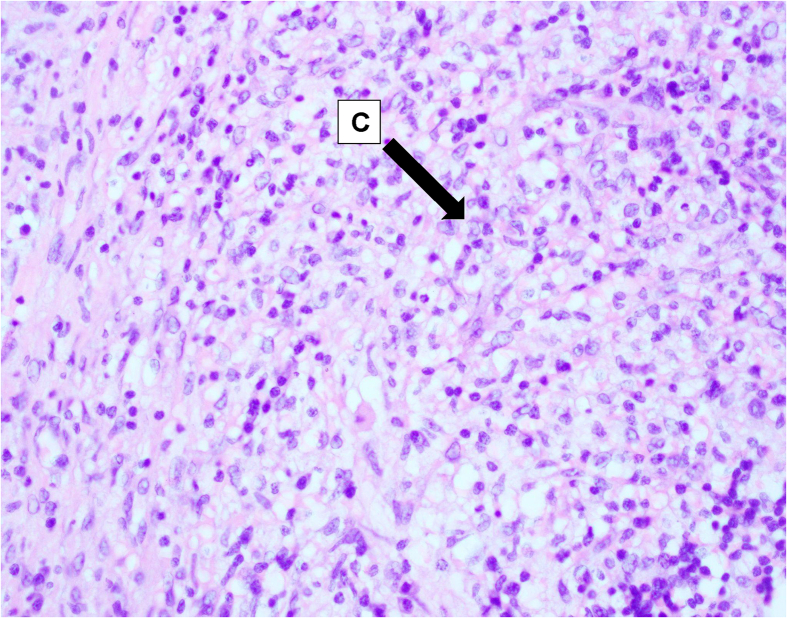
A-C: Excisional cervical lymph node biopsy: Well-circumscribed foci of necrosis with abundant karyorrhectic debris and scattered fibrin deposits. Lymphocytes and plasmacytoid monocytes are seen at the periphery of the necrotic zones. These findings are consistent with Kikuchi-Fujimoto Disease. A) Geographic necrosis B) Karyorrhectic debris. C) Lymphoplasmacytic infiltrates.

Bilateral discrete cervical lymph nodes at levels IIA/B, III, IV, and V were found to be enlarged. The largest cervical node measured 36 × 22 mm at level II on the left side. There were 4–5 prominent axillary nodes bilaterally, with the largest measuring 18 × 12 mm on the left side. Her excisional cervical lymph node biopsy revealed well-circumscribed foci of necrosis with abundant karyorrhectic debris and scattered fibrin deposits (Fig. 1 A-C). Lymphocytes and plasmacytoid monocytes were seen at the periphery of the necrotic zones (Fig. 1 A-C). At this point, a histological diagnosis of Kikuchi-Fujimoto Disease was established, and she was prescribed NSAIDs and discharged against medical advice after improvement in her condition.

### 2.2 Late presentation

**Fig. 2:**
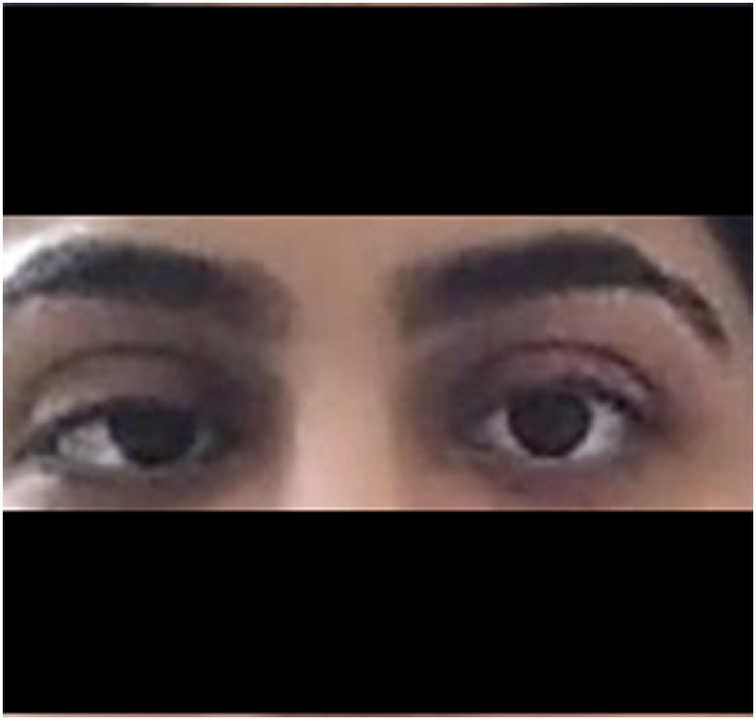
Sixth cranial nerve palsy leading to adduction of the right eye.

The patient presented again two weeks later with persistent fever (101 °F) accompanied by severe generalized headache, periorbital pain, double vision, and early morning vomiting. Her physical examination revealed right partial sixth cranial nerve palsy Fig. 2, no pupillary involvement, no signs of meningeal irritation, and no palpable lymph nodes this time. CBC again showed leukopenia with a WBC count of 2.5 × 10^9^/L (4–11 × 10^9^/L), microcytic hypochromic anemia with hemoglobin 10.0 g/dl, and a normal platelet count of 370 × 10^9^/L. Magnetic resonance imaging (MRI) of the brain with contrast was unremarkable. Subsequently, a cerebrospinal fluid (CSF) analysis was performed to assess for meningitis. The detailed findings of CSF analysis are shown in Table 1. CSF culture showed no microbial growth and Ziehl Neelsen (ZN) staining was negative. Based on CSF findings, the patient was diagnosed with aseptic meningitis and treated with intravenous pulse therapy of methylprednisolone (a total dose of 2.5 g). This was followed by oral prednisone 45 mg/day (0.75 mg/kg/day) for two weeks which was gradually tapered off over the next three months with the addition of hydroxychloroquine 200 mg/day to prevent relapse. The patient showed significant improvement in her symptoms within a week of initiating steroid pulse therapy and fully recovered soon after. The patient was, however, lost to follow-up, which hindered any attempt to determine prognosis.

**Table 1:** Result of CSF analysis.⁎⁎

Parameter (unit)	Value
LDH (U/L)	11
Glucose (mg/dl)	75
Protein (mg/dl)	20.1
RBCs (/cmm)	30
WBCs (/cmm)	10
Neutrophils (%)	10
Lymphocytes (%)	90

⁎⁎ Table 1 Abbreviations: White Blood Cells (WBCs), Red Blood Cells (RBCs), Lactate Dehydrogenase (LDH).

### 2.3 Differential diagnosis

The major differentials during the early presentation included tuberculosis, lymphoma, KFD, and SLE lymphadenitis. Histological results excluded these diagnoses and confirmed KFD. The subsequent presentation with aseptic meningitis was attributed to KFD based on a clear temporal relationship, knowledge of the treating physicians, and increasingly growing literature on this neurologic manifestation. The rapid response to steroids also supported this diagnosis.

## 3 Discussion

Kikuchi-Fujimoto Disease (KFD) was first reported in Japan in 1972, by Kikuchi and Fujimoto independently [7]. However, soon after its recognition as a novel clinical entity, cases were reported from other parts of the world as well [8]. KFD is primarily an inflammatory disease characterized by necrosis of the cervical lymph nodes, most commonly presenting with fever and unilateral cervical lymphadenopathy. However, variable presentations have been identified in the literature, including B-type symptoms such as weight loss, night sweats, fatigue, headache, and arthralgias [9].

The etiology of KFD is an unsolved conundrum, owing partly to the rarity, and partly to the non-specific nature of the disease itself. However, two models of pathogenesis have attempted to explain the development of this rare disorder. The first model proposes that KFD occurs due to an infectious process of microbial etiology. Several different viruses, including Epstein Barr Virus, Human Immunodeficiency Virus, Herpes viruses, Varicella-Zoster, etc., as well as bacteria such as Brucella, Bartonella, and Yersinia, have been suggested to directly cause KFD [10]. This theory derives its rationale from the clinical presentation of patients with KFD, which complements the diagnosis of a viral infection. For instance, the B symptoms, the lymph node histopathological findings, as well as a lack of response to antibiotics, all point toward a viral etiology [2]. However, the lack of identification of any infectious agent in most cases of KFD precludes the establishment of a definitive causation relationship. A second model of pathogenesis proposes an SLE-like self-limited small-scale autoimmune reaction [3] that drives the apoptosis of cluster of differentiation 8+ (CD8+) T cells in the affected lymph nodes [11], and thus explains the histological basis of the disease. However, the absence of any definitive autoimmune markers in serologic studies makes this model unreliable as well.

On account of the rarity of the disease, there exist no consensus guidelines regarding its treatment. It is believed that mild disease responds well to antipyretics and NSAIDs alone, while corticosteroids are needed for extra nodal manifestations such as aseptic meningitis [12]. Aseptic meningitis in our patient was treated with intravenous pulse therapy of methylprednisolone, later replaced with oral therapy which was gradually tapered off over 3 months. An improvement in symptoms was noticed within a week of starting this therapy, reiterating that prompt recognition and treatment of this complication can prevent neurological morbidity and mortality.

KFD is a rare disease worldwide and epidemiological data shows that Asia has a relatively high prevalence [13]. It is also imperative to note that KFD can present with a diverse nature of complications and poses a diagnostic dilemma, especially in tuberculosis endemic regions such as Pakistan where many a time it mimics tuberculous lymphadenitis and is thus not usually sought after by physicians. Presentation in young females raises a suspicion of SLE lymphadenitis and lymphoma, hence further complicating diagnosis. Central nervous system (CNS) involvement is rare in KFD and the few reported presentations include aseptic meningitis, encephalitis [14], optic neuritis, cerebellar ataxia and hemiplegia [15,16]. In 2006, Kucukardali et al. analyzed 244 published cases of KFD since 1991 and reported that neurological involvement was observed in only 4.5% of the patients [17]. In a more recent review by Dumas et al. [18] that analyzed 91 cases diagnosed between 1989 and 2011 in 13 French hospital centers, only 2 patients (2.2%) were found to have neurological manifestations. This affirms that the association of aseptic meningitis with KFD, an already rare disease, is a rare one and very few such cases have been reported globally [19]. However, to the best of our knowledge, no such case has ever been published from Pakistan.

Most of the published literature describes cases of KFD with aseptic meningitis as either its first manifestation or during an established course of KFD illness. [19,20]. However, our case report is unique in the fact that our patient showed non-specific neurological findings 2–3 weeks after treatment and resolution of KFD symptoms. Our patient was consistently negative for any signs of meningeal irritation and presented only with nonspecific (and nondiagnostic symptoms) such as headache and diplopia. This, coupled with a scarcity of published literature on KFD from this part of the world, leads to limited awareness among Pakistani clinicians regarding this unique association. This case report was however limited by a lost follow-up since the patient failed to report back after discharge, hindering any attempt to follow the prognosis.

## 4 Conclusion

Aseptic meningitis is a rare neurological sequela of Kikuchi-Fujimoto disease (KFD). The non-specific presentation of KFD, the absence of signs of meningeal localization, and limited available literature hinders physicians from recognizing this clinical association. It is important to note that meningitis may also develop after complete resolution of KFD symptoms, but if identified and treated promptly, most patients recover completely without any major morbidity.

## Patients' consent

Informed consent was taken from the patient for the publication of this case.

## Declaration of Competing Interest

Authors declare no conflicts of interest.
